# Job satisfaction among Syrian healthcare workers in refugee health centres

**DOI:** 10.1186/s12960-021-00685-x

**Published:** 2021-11-14

**Authors:** Monica Zikusooka, Omur Cinar Elci, Habibe Özdemir

**Affiliations:** 1 Refugee Health Programme, WHO Country Office in Turkey, WHO Regional Office for Europe, Ankara, Turkey; 2 SIHHAT Project, Migrant Health Department, Ministry of Health of the Republic of Turkey, Ankara, Turkey

**Keywords:** Job satisfaction, Healthcare, Health workers, Physicians, Refugee, Migrant, Nurses, Turkey

## Abstract

**Background:**

Achieving universal health coverage is subject to the availability, accessibility, acceptability, and quality of health workers. Countries that host refugees and migrants, such as Turkey, must strengthen the capacity of their health systems to increase access to services, especially for refugees and migrants. The Turkish Ministry of Health adapted Syrian refugee healthcare workers in the healthcare services to boost Syrian refugees’ access to healthcare. This study aimed to assess job satisfaction and the factors influencing job satisfaction among refugee physicians and nurses working in Refugee Healthcentres (RHCs) in Turkey.

**Methods:**

A self-administered, cross-sectional survey targeted all Syrian physicians and nurses working in RHCs across Turkey. The short-form Minnesota Satisfaction Questionnaire(MSQ) was used to assess job satisfaction. In total, 555 nurse/midwives and 336 physicians responded, yielding a total response rate of 56.5%. Descriptive analyses and linear regression tests were conducted to determine the level of job satisfaction and to analyze determinant factors.

**Results:**

Nurses/midwives reported the highest level of general job satisfaction, followed by specialist physicians and general physicians. Physicians who had worked as specialists in Syria but were now working as general physicians in Turkey had the lowest job satisfaction levels. Multiple regression analysis showed that professional status in Turkey, income, teamwork and team management were significantly associated with job satisfaction.

**Conclusions:**

To maintain a high level of job satisfaction in refugee healthcare workers, human resources management should consider matching job placements with training specialization and support good leadership and good teamwork. Remuneration that accounts for the cost of living and non-financial incentives could also play a significant role in job satisfaction.

## Introduction

Turkey hosts almost 3.7 million Syrian Refugees [1]. To meet the healthcare needs of the refugee population and to relieve pressure on the national health system workforce, the Ministry of Health (MoH) of the Republic of Turkey, established Refugee Healthcentres (RHCs), where Syrian healthcare workers (SHW) provide culturally and linguistically sensitive primary healthcare services. WHO, in collaboration with the MoH, implemented an adaptation training program to adapt qualified SHW to the Turkish healthcare system before employment in RHCs. SHW are authorized to work only in RHCs, where patients are only refugees or migrants, mainly Syrian refugees. RHCs may provide primary health services, such as vaccination, maternal and childcare services, and emergency health services to other migrant nationalities. Turkish nationals do not receive healthcare services from RHCs. Syrian nurses, midwives and general physicians work at the same level as they did in Syria, but according to regulatory procedures, specialist physicians are mainly employed as general physicians; only a small proportion continue to work as specialists. Syrian physicians in RHCs are paid the same wages as Turkish physicians working in primary healthcare services.

Refugee health workers face multiple challenges in continuing their profession while in host countries. Foremost is the lengthy and complex recognition process, which requires complex documentation and might face mismatched equivalency [2–5]. Yet, retraining is time, labor, and financially demanding against other family needs and stressors of migration [5–7]. As a result, some health workers may change to fields requiring fewer qualifications leading to underemployment [3, 8], a lost opportunity for the host country to address potential healthcare worker shortages and diversify the workforce [9]. Deskilling of healthcare workers disproportionally affects women [10]. Unable to continue their profession, health workers face professional disappointment and psychological effects [11, 12]. Some countries offer training and placement programs to fast track the integration of refugees [3, 7, 13, 14]; however, their successes are diffident [12–14]. Refugee and migrant health workers also face other barriers ranging from unfamiliarly with the health care system [15], different working cultures, and discrimination from patients and co-workers [3, 4]. Notwithstanding, refugee and other migrant healthcare workers present a valuable health workforce in host countries [16, 17].

Studies have shown that physicians’ satisfaction with their work and work environment may impact the quality of healthcare [18], patients’ satisfaction with their healthcare [19–22], patients’ adherence to medical instructions [23] and physicians’ commitment and retainment [18, 24]. The higher turnover rates for physicians due to poor job satisfaction may disrupt care provision and access to healthcare, while recruitment and replacement efforts may increase healthcare costs. Some factors affecting job satisfaction relate to the individual, such as age, gender, marital status, and work experience, while others are intrinsic to the nature of the job, such as specialization, patient interactions, and work engagement [25]. Yet, other factors can relate to the job context, such as workload, job security, and income level, or the work environment, such as the type of facility, management, professional development, teamwork, and access to resources [25]. Job satisfaction among nurses may be influenced by the work environment, workload, team support, stress and emotional exhaustion and ethnicity [26–29]. Job satisfaction among nurses has also been negatively associated with turnover [24, 27, 30].

Not as many studies have investigated job satisfaction among refugee healthcare workers as among migrant healthcare workers. Moreover, existing evidence on refugee and migrant healthcare workers largely relates to integration into the host country’s health system to provide services to the host population. However, in a unique structure in the Turkish health system, Syrian refugee healthcare workers provide culturally and language-sensitive services only to the Syrian refugee population through the RHCs mechanism. Although there might be similarities between the experiences of Syrian refugee healthcare workers in Turkey and refugee healthcare workers elsewhere, understanding job satisfaction in this unique context could contribute to human resource planning in countries with large refugee and migrant populations. This study sought to establish job satisfaction and identify the factors that determine job satisfaction among Syrian physicians and nurses working in RHCs in Turkey.

## Method

Data was collected using a self-administered, cross-sectional survey that targeted all Syrian physicians and nurses working in RHCs across Turkey. Job satisfaction was assessed using the General Job Satisfaction scale based on the short-form MSQ [31]. After reviewing various other tools to measure job satisfaction, short-form MSQ fit best with the work structure, job descriptions, and the aim of the study. The short-form MSQ included 20 items on job satisfaction: ability utilization, achievement, activity, advancement, authority, company policies, compensation, co-workers, creativity, independence, moral values, recognition, responsibility, security, social status, social service, supervision—human relations, supervision—technical, variety and working conditions. The determinants of job satisfaction were assessed using a separate questionnaire that was developed following a review of WHO field assessments in Turkey supported by a literature review on factors influencing job satisfaction in healthcare workers. The final combined questionnaire was translated from English to Arabic and then retranslated to English for consistency in the meaning of the questions. The final Arabic questionnaire was pretested to check the suitability of the Syrian Arabic dialect, clarity of content, and interpretation of questions.

### Sample and data collection

According to the MoH data, 681 Syrian physicians and 896 nurses were employed in RHCs across Turkey at the time of the survey. Between October and November 2019, an electronic link to the online questionnaire was sent to all physicians and nurses working in RHCs. The Department of Migration, MoH, through the medical directors in each RHC, sent weekly reminders to the healthcare workers for their participation. In total, 555 nurse/midwives and 336 physicians, giving an overall response rate of 56.5% (61.9% for nurses and 49.3% for physicians), completed the questionnaire.

### Data analysis

A descriptive analysis was conducted to summarize the distribution of the study population and determine the level of job satisfaction. Due to the non-parametric nature of the data, the inferential relationship between variables and the job satisfaction score was assessed using the Mann–Whitney U and the Kruskal–Wallis tests. For variables that had three groups, Dunn’s pairwise tests with adjustment using Bonferroni correction were conducted after the Kruskal–Wallis test to identify groups with significantly different levels of job satisfaction. Linear regression analysis was also conducted to determine the factors that influenced job satisfaction. Data were analyzed using IBM SPSS Statistics version 25.0.

## Results

Most of the participants were nurses/midwives (62%). Approximately two-thirds of respondents were men (65.2%), 56.7% were younger than 40, and 86.9% were married. Mostly physicians, 47.3% of participants were Syrians with Turkish citizenship (Table 1). Although most of the healthcare workers had an undergraduate degree or higher, for 28.8% of nurses, the highest education level was high school. Almost half (41.2%) of study participants had five or fewer years of work experience in Syria, and 64.5% had worked in Turkish healthcare services for 2 years or less.

**Table 1:** Sociodemographic and occupational characteristics of respondents

Characteristic	General physician (*n* = 259)		Specialist physician (*n* = 77)		Nurse/midwife (*n* = 555)		Total (*n* = 891)	
	*n*	%	*n*	%	*n*	%	*n*	%
Sex								
Male	209	80.7	58	75.3	314	56.6	581	65.2
Female	50	19.3	19	24.7	241	43.4	310	34.8
Age group (years)								
20–29	41	15.8	0	0.0	127	22.9	168	18.9
30–39	100	38.6	24	31.2	213	38.4	337	37.8
40–49	56	21.6	34	44.2	159	28.6	249	27.9
≥ 50	62	23.9	19	24.7	56	10.1	137	15.4
Nationality								
Syrian	111	42.9	31	40.3	328	59.1	470	52.7
Syrian with Turkish citizenship	148	57.1	46	59.7	227	40.9	421	47.3
Education								
High school	0	0.0	0	0.0	160	28.8	160	18.0
Undergraduate degree	166	64.1	16	20.8	386	69.5	568	63.7
Masters/postgraduate degree	93	35.9	61	79.2	9	1.6	163	18.3
Profession in Syria								
General physician	184	71.0	0	0.0	0	0.0	184	20.7
Specialist physician	75	29.0	77	100.0	0	0.0	152	17.1
Nurse/midwife	0	0.0	0	0.0	555	100.0	555	62.3
Years worked in Syria								
0–5	134	51.7	20	26.0	213	38.4	367	41.2
6–10	53	20.5	21	27.3	123	22.2	197	22.1
> 10	72	27.8	36	46.8	219	39.5	327	36.7
Years worked in Turkey^a^								
0–2	172	66.4	42	54.5	361	65.0	575	64.5
> 2	87	33.6	35	45.5	194	35.0	316	35.5

^a^In the Ministry of Health project

### Work characteristics and work environment

Almost half of the nurses/midwives (46.1%) reported seeing an average of 21–40 patients per day; in comparison, the highest proportions of both general and specialist physicians reported seeing more than 61 patients per day (46.7% and 39.0%, respectively) (Table 2). Most participants reported having access to the resources required for their work and knowing how to use the equipment and materials at their disposal. Among all participants, 88.2% rated the level of teamwork as good. A slightly lower proportion of all participants (77.7%) rated their team management as good; the lowest proportion of participants giving this assessment were general physicians (68.0%). Of the three professional groups, nurses were the most positive about their teamwork and team management. Overall, 46.1% of participants rated their income as average; this rating was reflected among the specialist physicians and nurses, while the highest proportion of general physicians assessed their income as poor.

**Table 2:** Work characteristics and work environment of respondents

Characteristic	General physician (*n* = 259)			Specialist physician (*n* = 77)		Nurse/midwife (*n* = 555)		Total (*n* = 891)	
	*n*		%	*n*	%	*n*	%	*n*	%
Type of health centre									
RHTC		23	8.9	11	14.3	42	7.6	76	8.5
RHC		215	83.0	56	72.7	491	88.5	762	85.5
Extended RHC		21	8.1	10	13.0	22	4.0	53	5.9
Average number of patients/day									
< 21	3		1.2	3	3.9	68	12.3	74	8.3
21–40	40		15.4	18	23.4	256	46.1	314	35.2
41–60	95		36.7	26	33.8	153	27.6	274	30.8
≥ 61	121		46.7	30	39.0	78	14.1	229	25.7
Access to resources needed for work									
Yes	196		75.7	49	63.6	489	88.1	734	82.4
No	63		24.3	28	36.4	66	11.9	157	17.6
Know how to use equipment/other materials									
Yes	256		98.8	77	100.0	551	99.3	884	99.2
No	3		1.2	0	0.0	4	0.7	7	0.8
Teamwork									
Poor	11		4.2	2	2.6	8	1.4	21	2.4
Average	34		13.1	7	9.1	43	7.7	84	9.4
Good	214		82.6	68	88.3	504	90.8	786	88.2
Team management									
Poor	34		13.1	8	10.4	27	4.9	69	7.7
Average	49		18.9	15	19.5	66	11.9	130	14.6
Good	176		68.0	54	70.1	462	83.2	692	77.7
Income									
Poor	118		45.6	23	29.9	73	13.2	214	24.0
Average	111		42.9	43	55.8	257	46.3	411	46.1
Good	30		11.6	11	14.3	225	40.5	266	29.9

### Job satisfaction

**Fig. 1:**
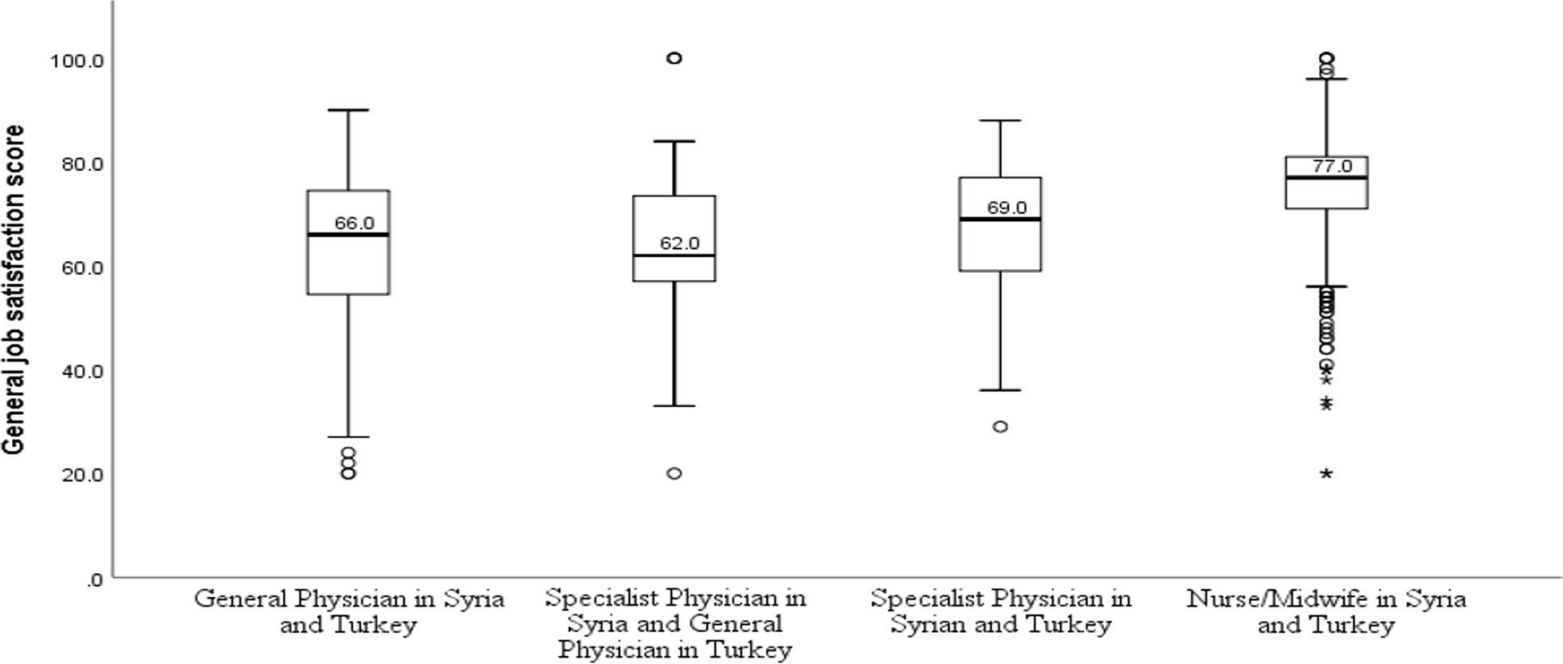
Job satisfaction level by profession and country of work

Nurses/midwives reported the highest level of general job satisfaction, followed by specialist physicians and then general physicians (Table 3). Kruskal–Wallis analysis provided strong support that job satisfaction levels differed between the three professional groups (*p* < 0.001); a follow-up Dunn’s pairwise test revealed that job satisfaction scores were significantly higher in nurses than in physicians, both general and specialist (*p* < 0.001). Further analysis revealed that physicians who had worked as specialists in Syria but were now working as general physicians in Turkey had the lowest satisfaction levels (Fig. 1). However, the difference in job satisfaction between sub-groups of physicians was not significant.

**Table 3:** Job satisfaction by profession

Profession	Participants (*n*)	Mean	SD	95% CI	Range
General physician	259	63.3	14.6	61.5–65.1	20.0–100.0
Specialist physician	77	65.9	13.2	63.2–69.0	29.0–88.0
Nurse/midwife	555	74.9	11.0	74.0–75.8	20.0–100.0
Total	891	70.8	13.4	69.9–71.7	20.0–100.0

**Fig. 2:**
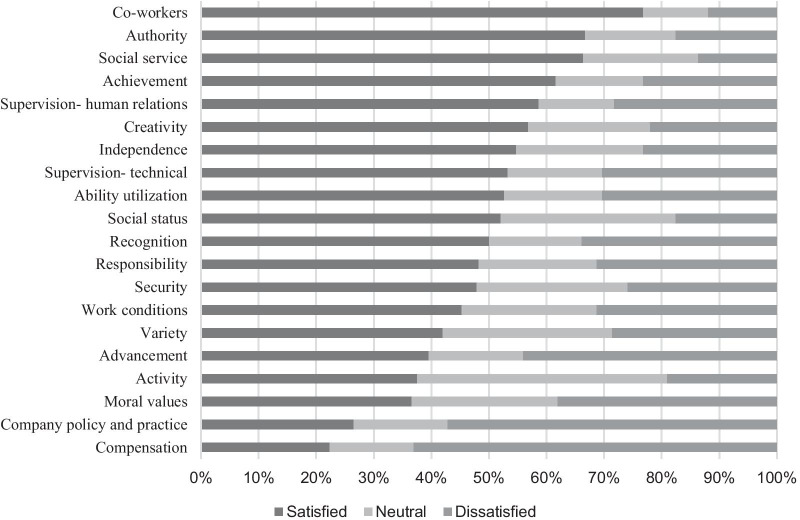
Levels of satisfaction related to the 20 items of the MSQ: physicians

**Fig. 3:**
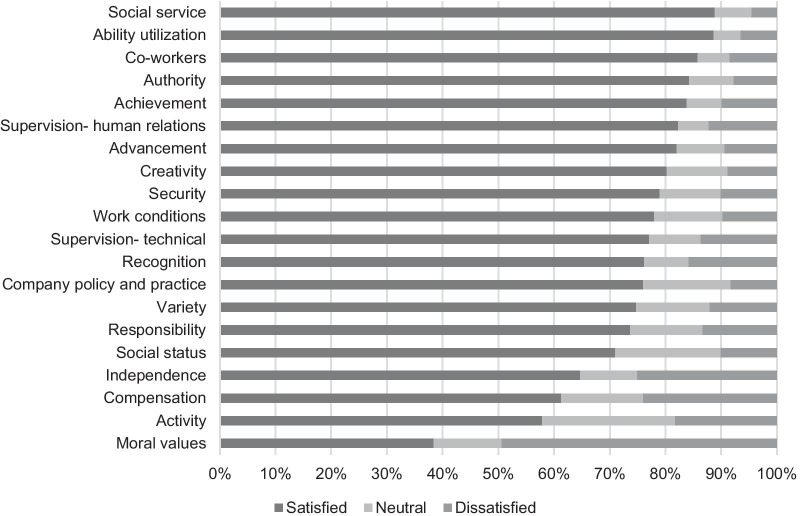
Levels of satisfaction related to the 20 items of the MSQ: nurses

As there was no significant difference in job satisfaction between general and specialist physicians, data for both groups were combined in the analysis of satisfaction on the items of the MSQ. Levels of satisfaction on the 20 items on the MSQ also varied among the different groups of participants (Figs. 2 and 3). Responses for the five-point Likert scale were combined into three categories: satisfied (satisfied and very satisfied), neutral and dissatisfied (dissatisfied and very dissatisfied). Physicians were most satisfied with the co-workers, authority and social service items and least satisfied with the compensation, workplace policy/practice and moral values items. Nurses were most satisfied with the social services, professional ability utilization, and co-workers and least satisfied with the moral values, compensation, and independence items. Overall, physicians were more dissatisfied than nurses with compensation.

### Factors affecting job satisfaction

#### Sociodemographic factors

Sociodemographic factors included age, gender, education, and Turkish citizenship. A statistically significant difference was found in the mean general job satisfaction score between age groups (*p* < 0.003), education levels (*p* < 0.001), and citizenship categories (*p* = 0.002). The mean general job satisfaction score was highest among those aged 40–49 years. When analyzed by education level, job satisfaction was highest among those with high school education. Job satisfaction scores were higher for healthcare workers without Turkish citizenship than those with Turkish citizenship (*p* = 0.002).

#### Work characteristics

The work characteristics assessed in this study were the number of patient consultations per day, access to resources (e.g., equipment and supplies) needed for, work and knowing how to use the available equipment and other materials. Job satisfaction scores were significantly different according to the number of consultations per day. Scores were highest among health workers who saw fewer than 21 patients per day (*p* < 0.001). There was a significant inverse relationship between the number of patients seen per day and job satisfaction scores (*p* < 0.001). Participants who reported having access to necessary resources also had significantly higher job satisfaction scores (*p* < 0.001).

#### Work environment

The factors used to assess the work environment of Syrian physicians and nurses were type of healthcentre and the participants’ perceptions of teamwork and team management. Job satisfaction scores were significantly influenced by all three factors. The mean job satisfaction score was significantly lower for those working in extended RHCs (*p* = 0.007) and significantly higher among those who rated teamwork and team management as good. Those who rated teamwork as poor had job satisfaction scores 13 points lower than those who rated it as good and three points lower than those who rated it as average (*p* < 0.001).

#### Profession, experience and income

The mean job satisfaction scores were significantly higher in nurses than in the physicians (*p* < 0.001). Job satisfaction scores also differed significantly by the number of years they worked in Syria (*p* < 0.001) but not by the number of years working in Turkey (*p* = 0.190). Compared with the other categories for years worked in Syria, participants with over 10 years of experience had the highest mean job satisfaction scores. The mean job satisfaction score was directly associated with the perception of income earned from working in the RHCs (from poor to good; *p* < 0.001). Compared with those who rated their income as good, the job satisfaction score was 7.7 points lower among those who rated it as poor and 3.4 points lower among for those who rated it as average.

Multiple linear regression analysis was used to determine which factors likely affected job satisfaction. Variables included in the analysis were age group, nationality, number of patients per day, type of health centre, teamwork, team management, job in Turkey, number of working years in Syria and income. Out of these factors, only profession in Turkey and perception of income, teamwork and team management were significantly associated with job satisfaction (Table 4).

**Table 4:** Multiple regression analysis of factors associated with the general job satisfaction score

Variable	*B*	95% CI		SE	*p* value
		Lower bound	Upper bound		
Age group (years) (ref. ≥ 50)					
20–29	− 4.32	− 7.53	− 1.12	1.634	0.008*
30–39	− 4.61	− 7.28	− 1.94	1.361	0.001*
40–49	− 0.48	− 2.59	1.62	1.070	0.651
Nationality (ref. Syrian with Turkish citizenship)	0.32	− 0.97	1.61	0.655	0.626
Education (ref. High school)					
Undergraduate degree	− 0.52	− 2.45	1.42	0.984	0.600
Masters/postgraduate degree	− 0.31	− 3.12	2.50	1.432	0.829
Number of patients/day (ref. < 20)					
21–40	− 0.81	− 3.26	1.63	1.247	0.515
41–60	− 0.45	− 2.97	2.07	1.285	0.725
≥ 61	− 1.52	− 4.19	1.15	1.361	0.265
Type of health centre (ref. RHTC)					
RHC	− 1.65	− 3.93	0.63	1.161	0.156
Extended RHC	− 2.00	− 5.38	1.38	1.721	0.245
Teamwork (ref. Good)					
Poor	− 13.44	− 18.11	− 8.77	2.379	0.000*
Average	− 3.82	− 6.13	− 1.51	1.178	0.001*
Team management (ref. Good)					
Poor	− 15.85	− 18.63	− 13.08	1.412	0.000*
Average	− 9.31	− 11.27	− 7.35	0.998	0.000*
Profession (ref. Nurse/midwife)					
General physician	− 6.84	− 8.81	− 4.88	1.000	0.000*
Specialist physician	− 6.60	− 9.59	− 3.62	1.522	0.000*
Years worked in Syria (ref. > 10)					
0–5	1.99	− 0.37	4.35	1.202	0.099
6–10	2.26	0.05	4.48	1.130	0.045*
Income (ref. Good)					
Poor	− 7.79	− 9.77	− 5.81	1.009	0.000*
Average	− 3.24	− 4.77	− 1.72	0.777	0.000*

*p < 0.05

## Discussion

SHW in RHCs provide a critical human resource in response to the healthcare needs of 3.6 million Syrian refugees in Turkey. Previous field assessments among SHW showed that physicians and nurses valued the opportunity to work in Turkey and continuing to work in their profession [32]. They also appreciated the opportunity to earn their livelihood and serving their fellow Syrian refugees [32].

This study found that the job satisfaction levels were higher in nurses than in physicians. After completing adaptation training, Syrian nurses continue to work as nurses in RHCs but perhaps the more advanced health system in Turkey provides more opportunities to apply their skills, increasing job satisfaction. In comparison, physicians working in RHCs provide only primary healthcare services, limiting their ability to use their skills, especially for specialist physicians. Physicians who had trained and worked as a specialist in Syria but were working as general physicians in Turkey had a lower job satisfaction than those who continued to work as specialists in Turkey. Although the difference between satisfaction scores in our study was not statistically significant, previous studies reported that specialization and work engagement were associated with job satisfaction [23, 33]. Indeed, the profession in Turkey was a significant factor for job satisfaction for healthcare workers in RHCs. The RHCs provide a time-bound response to health service provision to Syrian refugees in Turkey. However, there is no mechanism for professional advancement for SHW; unsurprisingly, physicians expressed dissatisfaction with the lack of opportunities for advancement. Other studies have demonstrated that opportunities for advancement affect staff motivation and job satisfaction among healthcare workers [34]. Improving professional engagement for physicians in RHCs could enhance their job satisfaction levels, motivation, and intention to stay on the job and positively impact job performance in terms of the effectiveness and quality of care and patient satisfaction.

The mean job satisfaction score increased with the perception of income adequacy. Most study participants said their income was average. Evidence of inadequate income was provided by the low rating for compensation by both nurses and physicians, but particularly by physicians. Income was a significant predictor of job satisfaction. A qualitative study on factors affecting the employability of SHW revealed that the cost of living, not having other salary supplements, the costs of transportation, lunch and child care, and not being allowed to supplement their work with private practice limited the adequacy of their income from RHCs [32]. These factors may explain the importance of income in determining job satisfaction in healthcare workers in RHCs. In addition, comparing their income with Turkish physicians working in hospitals and not those working in primary health care may skew their perception of income adequacy. Studies conducted elsewhere showed that remuneration influences job satisfaction [35] and that non-monetary incentives increased professional and performance satisfaction among physicians [22, 35]. In fact, one study argued that non-monetary incentives could be more important than monetary incentives in supplementing physicians’ incomes [36]. Annual cost of living adjustments to the salaries of healthcare workers in RHCs coupled with non-financial incentives could improve job satisfaction.

Physicians and nurses considered that co-workers get along with each other to a satisfactory extent, which indicates good teamwork at RHCs. The study also found that the quality of teamwork is a significant determinant of job satisfaction. Relationships between co-workers shape the work environment and influence job satisfaction in general. A previous study demonstrated that job satisfaction in the delivery of healthcare was predicted by organizational culture and teamwork [37]*.* Others found that collaboration between workers and collegial relationships were associated with job satisfaction [26, 37–39]. Therefore, maintaining good teamwork in RHCs is critical to maintaining a supportive working environment for the health workers.

Management and leadership are essential for delivering healthcare services. Poor management of planning and coordination processes, care processes, human resources, and information flow negatively impact the work environment and job satisfaction in healthcare workers. This study found that the quality of team management was significantly associated with job satisfaction. Compared with those who rated team management as good, job satisfaction levels were 15.9 points lower in healthcare workers who rated team management as poor and 9.3 points lower in those who rated it average. Other studies also found that the quality of leadership was positively associated with physician satisfaction [25, 40]. Another found an association between the quality of nurse management at the unit level and job satisfaction [26]. Therefore, strengthening leadership and management in RHCs may positively impact the job satisfaction of SHW; hence, good RHC management may lead to positive outcomes for health workers and the quality of health service delivery.

Patient workload may have an impact on the job satisfaction of health workers. A previous report found that the nursing workload and staffing correlate strongly with the quality of outcomes for patients, nurses, and the organization [41]. Other evidence indicated that increased patient workload or work-related stress could decrease job satisfaction for physicians [42, 43], which impacts individual and organizational performance, absenteeism, and turnover [42]. Similarly, in the present study, the mean job satisfaction score decreased with increasing patient load. The dissatisfaction may be explained by the difference in working hours between Syria and Turkey, with fewer working hours in Syria. However, when other factors were considered, the number of patients seen per day did not significantly affect job satisfaction, suggesting that, at least for this population, it may not be an important factor.

Job satisfaction was higher in health workers who did not yet have Turkish citizenship, but the association was not significant when other factors were considered. Acquiring citizenship might be expected to lead to greater job satisfaction, because it provides opportunities for integration into the labor market. However, the higher satisfaction levels of participants without Turkish citizenship might simply indicate a more positive response or information bias by these participants; therefore, this result should be interpreted cautiously.

Both nurses and physicians expressed dissatisfaction on the professional moral values item of the MSQ, but further examination is required to determine the explanation. However, differences in the working context might present the healthcare workers with issues that challenge the values they hold as foreigners.

This was the first study to examine job satisfaction among SHW in RHCs in Turkey. Including all SHW and the response of rate of 56.5% were major strengths of the study. A self-administered questionnaire and the possibility of information bias should be considered when interpreting the results. However, the overall data quality suggests that evidence obtained in the study can be used to improve job satisfaction in RHCs and makes a valuable addition to the body of knowledge on refugee healthcare workers. Although factors influencing job satisfaction were identified, these can only explain some of the variation in the job satisfaction score. Further research could determine other factors that influence job satisfaction among Syrian health workers. In addition, comparative studies of Turkish and Syrian health workers and studies on labor market integration of Syrian health workers could shade more light on challenges and opportunities for strengthening the health workforce. Researchers should also focus on the labor market dynamics and the close connection between the healthcare workers’ job satisfaction and patient satisfaction.

## Conclusions

This study showed that job satisfaction for SHW in Turkey was determined by profession in Turkey, income, teamwork, and team management. In this and other contexts with refugee healthcare workers, human resources management should consider developing evidence-informed policies to accommodate refugee healthcare workers with equity and balance, providing opportunities for matching job placements with training specialization of refugees, maintaining good leadership and teamwork, and remuneration that accounts for the cost of living coupled with non-financial incentives. Establishing an integrated, fair work environment could contribute to maintaining a high level of job satisfaction in refugee healthcare workers and, consequently, performance levels and patient satisfaction.

## Data Availability

The data sets generated and/or analyzed during the current study are available in the [WHO] repository.

## Abbreviations

CI: Confidence interval
MSQ: Minnesota Satisfaction Questionnaire
RHC: Refugee Healthcentre
RHTC: Refugee health trainingcentre
SD: Standard deviation
SIHHAT: Improving the health status of the Syrian population under temporary protection and related services provided by Turkish authorities (project)
